# Association Between Dietary Alcohol Intake and Migraine or Severe Headache Miscellaneous Pain: The NHANES 1999–2004

**DOI:** 10.1002/brb3.70400

**Published:** 2025-03-14

**Authors:** Yi Tang, Kangrui Zhang, Yueyu Zhang, Xinhui Jia, Jiaxuan Li, Jie Hu, Xun He, Xinyi Chen, Juncang Wu

**Affiliations:** ^1^ Department of Neurology Hefei Hospital Affiliated to Anhui Medical University (The Second People's Hospital of Hefei) Hefei China; ^2^ Department of Neurology, The Fifth Clinical Medical College of Anhui Medical University Hefei China; ^3^ Department of Neurology Hefei Second People's Hospital affiliated to Bengbu Medical University Hefei China

**Keywords:** dietary alcohol intake, migraine, NHANES, severe headache

## Abstract

**Background:**

The relationship between alcohol consumption and migraine or severe headache remains controversial in the existing literature. Given that alcohol is a widely consumed beverage, clarifying the relationship between alcohol and migraine or severe headaches can help manage the patient's condition.

**Aim:**

This study aimed to investigate the potential relationship between alcohol consumption and migraine or severe headache.

**Methods:**

Employing National Health and Nutrition Examination Survey (NHANES) database records spanning March 1999 to December 2004, our analysis encompassed threshold effects, smoothed curve fitting, and multivariate logistic regression to elucidate the relationship between alcohol consumption level and migraine or severe headaches. We utilized subgroup analyses and interaction tests to explore the stability of this relationship across different stratified populations.

**Results:**

A total of 13,083 subjects were enrolled. The odds of migraine or severe headache decreased with increasing dietary alcohol intake. This was more pronounced in the older and male subgroups.

**Conclusions:**

There was a significant negative association between dietary alcohol intake and the odds of having migraine or severe headache.

## Graphical abstract

This study shows that alcohol intake is negatively associated with the odds of having a migraine, which is influenced by age and gender. Although not universally contraindicated, limiting alcohol remains advisable due to broader health risks.

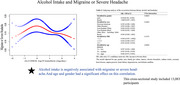

**Abbreviations**
GABA: gamma‐aminobutyric acid
NHANES: National Health and Nutrition Examination Survey
PIR: income‐to‐poverty ratio

## 1 Background

Epidemiological studies suggest that nearly all Americans will encounter headache issues at some stage in their lives (Rasmussen et al. 1991; Robbins 2021). In the United States, migraine is a common neurological disorder, affecting approximately 17% of women and 6% of men. Within the past 3 months, about 18% of American women reported experiencing severe headaches or migraine attacks (Chen et al. 2024). A study encompassing 162,756 individuals in the United States documented that 17.4% encountered intense cephalalgias conforming to the International Classification of Headache Disorders‐2 criteria, with 11.8% aligning with the diagnostic profile for migraine and 4.6% being identified as having likely migraine (Buse et al. 2013). Consequently, individuals experiencing severe headaches in the US are predominantly afflicted by migraine. Migraine, as a prevalent primary headache disorder, is characterized by episodic, lateralized, and moderate‐to‐severe pulsating pain (Raggi et al. 2024; Dodick 2018). In addition, emerging research in the United States has uncovered novel genetic indicators linked to migraine, thereby enhancing our comprehension of the underlying mechanisms of this condition (Bahrami et al. 2022; Grangeon et al. 2023; Zhang et al. 2025). Migraine is differentiated into two distinct subtypes: migraine with aura and migraine without aura (May 2018). Females exhibit a higher incidence of migraine compared to males (Stewart et al. 2008). Episodes of migraine are linked to a variety of both intrinsic and extrinsic precipitants. Endogenous factors are mostly associated with puberty and menstruation, suggesting that the onset of migraine is related to endocrine and metabolic factors. Exogenous factors, on the other hand, are related to factors such as food and drugs (Hindiyeh et al. 2020). Dietary aspects include nitrate‐containing meats, phenylethylamine‐containing chocolate, ice cream, tomatoes, onions, coffee, and other substances (Martin and Vij 2016a, 2016b; Cairns 2016). However, food triggers are likely individualized and cannot be generalized. In addition, migraine is not only disabling, especially among young adults and middle‐aged women, but also cause a serious economic burden, and therefore deserve our attention (Hu et al. 1999; GBD 2016 Headache Collaborators 2018; Waliszewska‐Prosół et al. 2024).

Alcohol is a widely consumed beverage globally, with some 2.3 billion people drinking nearly 35 billion liters of pure ethanol a year (Sohi et al. 2021). However, many health problems, such as cancer and disability, are strongly associated with alcohol intake (Safiri et al. 2022; GBD 2017 Cirrhosis Collaborators 2020). Even only light drinking (<12.5 g/day) increases the risk of oral and laryngeal cancers (Rumgay et al. 2021).

Current clinical and experimental studies are yet somewhat contradictory in terms of the relationship that exists between alcohol consumption and migraine or severe headaches (Lipton et al. 2014). Previous studies support alcohol consumption as a risk factor or precipitating factor for migraine (Hindiyeh et al. 2020; Zarshenas et al. 2013). However, the investigation of specific drinking patterns, such as frequency of drinking and specific dosage factors, was incomplete. In contrast, some literature concludes that alcohol consumption reduces the risk of headache occurrence (Błaszczyk et al. 2023; Yokoyama et al. 2012; Panconesi et al. 2013). The report noted that about 60%–80% of the patients who participated in the study consumed beer on 7%–10% of the days, which also reduced the risk of headaches and migraine (Wöber et al. 2007). This difference may be related to differences in drinking patterns, including the type of alcohol, frequency of drinking, and other factors (Yokoyama et al. 2012). Therefore, we need more evidence to explore the relationship between alcohol consumption and migraine or severe headache. In addition, analyzing exposure factors associated with migraine attacks will help us to delve deeper into the pathogenesis of migraine (Guidotti et al. 2013). This paper utilizes a cross‐sectional study design to explore the potential relationship that exists between dietary alcohol intake and migraine drawing on data extracted from National Health and Nutrition Examination Survey (NHANES) spanning 1999–2004.

## 2 Methods

### 2.1 Study Population

The NHANES constitutes a comprehensive research initiative designed to assess the dietary intake and overall health of the US population, encompassing both questionnaire responses and dietary assessments within its scope (Zhang et al. 2022; Xie and Zhang 2023). We used information from six consecutive NHANES survey phases, which ran from March 1999 to December 2004. Researchers were unable to obtain data that could reveal the identities of subjects throughout the data‐gathering process and subsequent analysis.

**FIGURE 1:**
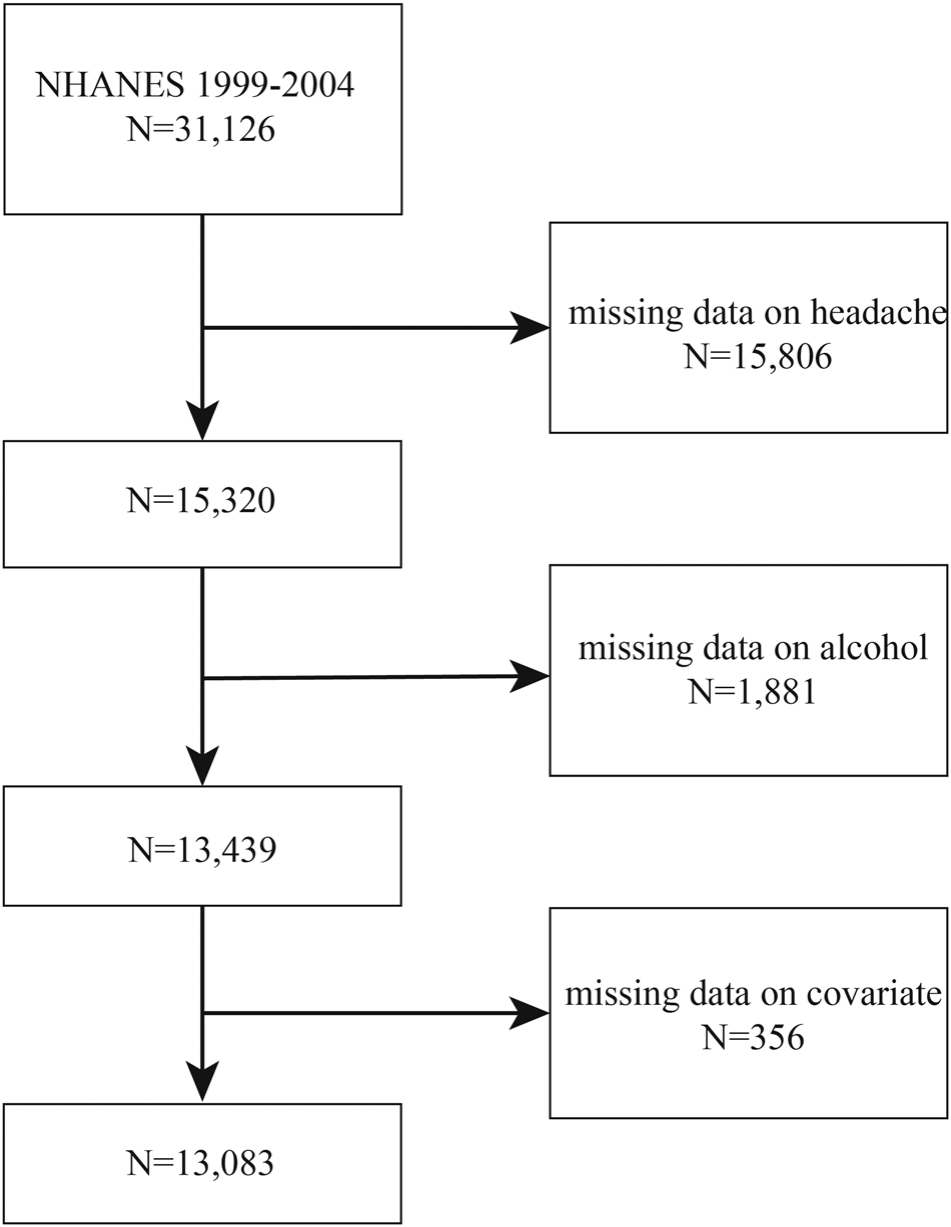
Flow chart of participants selection. Covariates that were censored included hypertension (*N* = 140), diabetes (*N* = 4), asthma (*N* = 14), heart failure (*N* = 58), coronary heart disease (*N* = 51), angina pectoris (*N* = 37), stroke (*N* = 11), chronic bronchitis (*N* = 25), and tumors (*N* = 16).

The total number of people eligible for the study was 31,126, of which 15,806 had missing headache data, 1881 had missing dietary alcohol intake, and another 356 were also excluded because of missing some covariates. Thirteen thousand and eighty‐three participants aged 20–85 with finished information on all independent and dependent variables were included (Figure 1).

### 2.2 Results and Exposure Factors

We determined whether participants had a history of migraine or severe headaches based on the results of the questionnaire (Peng et al. 2023). If “Have there been incidents of severe headaches or migraine episodes within the preceding quarter?” The answer to this question is “yes”, and we assume that the participant has a history of migraine or severe headaches. The categorization of people with severe headaches as having migraine is supported by the American Migraine Prevalence and Prevention study's findings (Buse et al. 2013). Other literature has taken the same hypothetical approach (Evans et al. 2015).

Our primary exposure factor was participants' exposure to alcohol in the Dietary Interview Total Nutrient Intake (DRXTOT). In this study, the total alcohol intake (mg) was obtained from 24‐h dietary recall interviews conducted by the Mobile Examination Center (MEC).

### 2.3 Covariables

To improve the stability of exposure factors and outcomes, we included the following variables: age, gender, ethnicity, income‐to‐poverty ratio (PIR), hypertension, diabetes, asthma, angina, heart failure, heart disease, stroke, chronic bronchitis, and cancer (Zhou et al. 2023). We divide ethnicity into five groups: Mexican American, other Hispanic, non‐Hispanic white, non‐Hispanic black, and other races. Gender is divided into male and female.

### 2.4 Statistical Analysis

Statistical evaluations in this study were performed using R (http://www.r‐project.org) and EmpowerStats (http://www.empowerstats.com) when *p* < 0.05 was considered statistically significant. We categorized alcohol intake (g/day) into three groups: A1 from 0 to 10, A2 from 10 to 150, and A3 greater than 150. This categorization allowed us to roughly assess the association between alcohol intake and the prevalence of migraine in low, medium, and high categories. In addition, we also applied multivariate logistic regression to explore the association between alcohol intake as a continuous variable and alcohol intake as a subgroup variable with migraine, respectively. We did not account for any confounders in Model 1. In Model 2, adjustments were made for gender, age, race, and PIR. In Model 3, we accounted for every covariate in Model 3, including gender, age, race, family PIR, hypertension, diabetes, coronary heart disease, angina, heart failure, chronic bronchitis, asthma, tumor, and stroke. We then transformed the logarithm of alcohol intake for continuous variables to do curve fitting to explore its nonlinear associations and used threshold effects analysis to calculate its inflection point K. In subgroup analyses, we used interaction tests to explore whether the relationship between alcohol intake and migraine was significant in subgroups of family PIR, gender, and age. We categorized age into three equal groups of low, medium, and high, while family pir was categorized into three groups using 1.3 and 3.5 as cutoff points.

## 3 Results

### 3.1 Baseline Characteristics

A total of 13,083 people met the inclusion criteria for this study, with an average age of 49.024 years and 47.08% being male. The average alcohol consumption in this study population was 9.643 mg/day and the prevalence of headache was 20.42%. As shown in Table 1, we categorized dietary alcohol intake (mg/day) into three groups: A1 for intake less than 10 mg/day, A2 for 10–150 mg/day, and A3 for more than 150 mg/day. The results show that the group with high alcohol intake was younger than the group with low alcohol intake. Additionally, age decreased with increasing alcohol intake. In addition, women consume less alcohol than men, with a decreasing number of women in all three groups.

**TABLE 1:** Basic characteristics of participants by dietary alcohol intake in US adults.

	Dietary alcohol intake (mg/day)			
Characteristics	A1	A2	A3	*p* value
*N*	10,572	2404	107	
**Family PIR**	2.501 ± 1.519	3.023 ± 1.583	2.655 ± 1.644	<0.001
**Age (months)**	594.747 ± 222.075	564.843 ± 199.759	491.140 ± 168.828	<0.001
**Gender**				<0.001
Male	4437 (41.969%)	1637 (68.095%)	86 (80.374%)	
Female	6135 (58.031%)	767 (31.905%)	21 (19.626%)	
**Race**				<0.001
Mexican American	2424 (22.928%)	442 (18.386%)	16 (14.953%)	
Other Hispanic	505 (4.777%)	86 (3.577%)	5 (4.673%)	
Non‐Hispanic White	5230 (49.470%)	1369 (56.947%)	57 (53.271%)	
Non‐Hispanic Black	2032 (19.221%)	445 (18.511%)	25 (23.364%)	
Other race—including multi‐racial	381 (3.604%)	62 (2.579%)	4 (3.738%)	
**Tumor**				0.123
Yes	947 (8.958%)	194 (8.070%)	5 (4.673%)	
No	9625 (91.042%)	2210 (91.930%)	102 (95.327%)	
**Chronic bronchitis**				0.387
Yes	645 (6.101%)	131 (5.449%)	8 (7.477%)	
No	9927 (93.899%)	2273 (94.551%)	99 (92.523%)	
**Stroke**				<0.001
Yes	397 (3.755%)	45 (1.872%)	2 (1.869%)	
No	10,175 (96.245%)	2359 (98.128%)	105 (98.131%)	
**Angina pectoris**				<0.001
Yes	418 (3.954%)	55 (2.288%)	2 (1.869%)	
No	10,154 (96.046%)	2349 (97.712%)	105 (98.131%)	
**Heart disease**				0.001
Yes	493 (4.663%)	76 (3.161%)	1 (0.935%)	
No	10,079 (95.337%)	2328 (96.839%)	106 (99.065%)	
**Asthma**				0.204
Yes	1184 (11.199%)	244 (10.150%)	15 (14.019%)	
No	9388 (88.801%)	2160 (89.850%)	92 (85.981%)	
**Heart failure**				<0.001
Yes	368 (3.481%)	37 (1.539%)	1 (0.935%)	
No	10,204 (96.519%)	2367 (98.461%)	106 (99.065%)	
**Diabetes**				<0.001
Yes	1172 (11.086%)	112 (4.659%)	4 (3.738%)	
No	9250 (87.495%)	2271 (94.468%)	102 (95.327%)	
Borderline	150 (1.419%)	21 (0.874%)	1 (0.935%)	
**Hypertension**				<0.001
Yes	3485 (32.964%)	637 (26.498%)	24 (22.430%)	
No	7087 (67.036%)	1767 (73.502%)	83 (77.570%)	
**Migraine or severe headache**				<0.001
Yes	2325 (21.992%)	333 (13.852%)	14 (13.084%)	
No	8247 (78.008%)	2071 (86.148%)	93 (86.916%)	

*Notes*: A1: 0 ≤ Alcohol < 10; A2: 10 ≤ Alcohol < 150; A3: 150 ≤ Alcohol(mg/day).

Results in table: Mean + SD/*N* (%).

*p* value: Kruskal–Wallis rank sum test for continuous variables, Fisher's exact probability test for count variables with theoretical number <10.

Abbreviation: PIR, income‐to‐poverty ratio.

### 3.2 Association Between Alcohol and Headache

**FIGURE 2:**
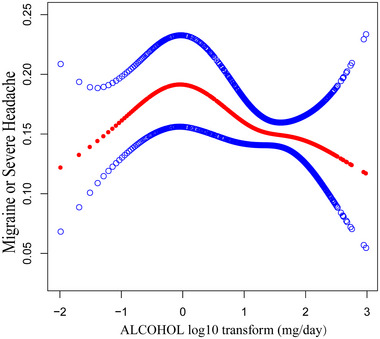
Non‐linear relationship between the transformation of alcohol log10 and headache. The red line depicts the smooth curve of the log10‐transformed alcohol and headache relationship. The blue lines illustrate the 95% confidence interval. A GAMM revealed a nonlinear link between alcohol and headache.

Data presented in Table 2 illustrate that across the trio of analytical models, there is a consistent observation of a negative association between the ingestion of alcohol in the diet and the odds of migraine or severe headaches. Upon adjusting for all confounding variables, a one‐unit increase in alcohol intake in model 3 was associated with a 0.4% lower in the odds of migraine or severe headaches (OR = 0.996, 95% CI: 0.994, 0.998). This association became more pronounced after categorization, with a 31.7% decrease in the odds of migraine or severe headaches for subjects in A2 (OR = 0.683, 95% CI: 0.599, 0.780) and a 46% decrease in A3 (OR = 0.540, 95% CI: 0.301, 0.968). Furthermore, the smoothing curve fitting (Figure 2) indicates a nonlinear negative association between alcohol consumption and migraine. We calculated the inflection point *k* = −0.097 using threshold effect analysis. It was not statistically significant (OR = 1.412, 95% CI: 0.976, 2.043) on the left side of inflection point *k* and statistically significant (OR = 0.801, 95% CI: 0.662, 0.968) on the right side. This suggests that, to the right of turning point *k*, alcohol consumption is negatively associated with migraine.

**TABLE 2:** Association between dietary alcohol (g/day) and headache.

Exposure	Model 1 OR (95% CI), *p* value	Model 2 OR (95% CI), *p* value	Model 3 OR (95% CI), *p* value
Alcohol (continuous)	0.994 (0.992, 0.996) <0.00001	0.996 (0.994, 0.998) 0.00002	0.996 (0.994, 0.998) 0.00002
Alcohol			
A1	1.0	1.0	1.0
A2	0.570 (0.504, 0.646) <0.00001	0.677 (0.594, 0.771) <0.00001	0.683 (0.599, 0.780) <0.00001
A3	0.534 (0.304, 0.938) 0.02916	0.575 (0.323, 1.021) 0.05906	0.540 (0.301, 0.968) 0.03851

*Notes*: A1: 0 ≤ Alcohol<10; A2: 10 ≤ Alcohol<150; A3: 150≤ Alcohol.

Model 1: did not adjust for covariates.

Model 2: adjusted for age, gender race family PIR values.

Model 3: adjusted for age, gender race family PIR values, tumors, chronic bronchitis, asthma stroke, coronary heart disease, angina pectoris, heart failure, hypertension, and diabetes mellitus.

Abbreviations: CI, confidence intervals; OR, odds ratio.

### 3.3 Subgroup Analyses

In this study, subgroup analyses of gender, age, race, and family PIR were conducted, thus ensuring that the relationship between dietary alcohol intake and headache remained stable across demographic contexts. As shown in Table 3, family PIR and race failed to significantly influence the relationship between alcohol and migraine or severe headache (*p* > 0.05). However, we could observe that gender altered this relationship (*p* = 0.0023). The odds of migraine were 0.7% lower in the male population, while no such correlation was observed in women. In addition, there was a difference between the different age subgroups (*p* = 0.0094). Compared to the younger age group (OR = 0.997, 95% CI: 0.994, 1.000), the older age group had 1.5% lower odds of migraine (OR = 0.982, 95% CI: 0.971, 0.992).

**TABLE 3:** Subgroup analysis of the association between dietary alcohol and headache.

	OR(95% CI)	*p* for interaction
**Stratified by gender**		0.0023
Male	0.993 (0.991, 0.996)	
Female	1.000 (0.997, 1.003)	
**Stratified by race**		0.6863
Mexican American	0.995 (0.990, 1.000)	
Other Hispanic	0.995 (0.986, 1.005)	
Non‐Hispanic White	0.996 (0.994, 0.999)	
Non‐Hispanic Black	0.996 (0.992, 1.000)	
Other race—including multi‐racial	0.985 (0.969, 1.002)	
**Stratified by PIR**		0.2521
<1.3	0.998 (0.995, 1.000))	
1.3–3.5	0.994 (0.990, 0.997)	
≥3.5	0.995 (0.991, 0.999)	
**Stratified by age (trisect)**		0.0094
T1: Low	0.997 (0.994, 1.000)	
T2: Medium	0.995 (0.992, 0.998)	
T3: High	0.982 (0.971, 0.992)	

*Notes*: This model adjusted for age, gender, race, family PIR values, tumors, chronic bronchitis, asthma, stroke, coronary heart disease, angina pectoris, heart failure, hypertension, and diabetes mellitus.

Abbreviations: CI, confidence intervals; OR, odds ratio; PIR, income‐to‐poverty ratio.

## 4 Discussion

In this cross‐sectional study of 13,083 participants, we observed a negative association between alcohol consumption and the odds of migraine or severe headaches. Age and gender had a significant effect on this correlation. Furthermore, in a study by Mostofsky et al., it was found that drinking 1–2 servings of alcohol may not have a significant effect on headache; whereas drinking five or more servings of alcohol may increase the risk of headache (Rasmussen 1993). A similar threshold effect was observed in our study, with a significant decreasing trend in the odds of migraine or severe headache to the right of the turning point *k* (−0.097) (Figure 2).

There are discrepancies in current research regarding the relationship between alcohol consumption and migraine or severe headaches. A previous prospective observational study in the Korean region included 62 participants. The subjects kept diaries until the end of the study, and the researchers analyzed 4579 days of diary data, which showed that many factors, including alcohol, increased the risk of migraine (Park et al. 2016). Such results tend to favor that alcohol is a trigger for migraine attacks. In contrast, alcohol consumption was linked to a 1.5‐fold lower risk of migraine, according to a study with a sample size of over 100,000 people (Błaszczyk et al. 2023; Yokoyama et al. 2012).

One possible explanation is that migraine patients tend to avoid alcohol (Rafi et al. 2022). This may be related to several factors. First, a significant number of migraine patients with aura who are sensitive to alcohol reject alcohol (Karli et al. 2005). Studies from China have reported that cluster headache patients reduce their alcohol consumption during clusters (Lin et al. 2004; Dong et al. 2013; Xie et al. 2013). Second, the alcohol dehydrogenase‐1B (ADH1B) encoded by the ADH2*2 allele is more active, metabolizes alcohol faster, and produces large amounts of acetaldehyde more rapidly during alcohol consumption (García‐Martín et al. 2010). Whereas the production of acetaldehyde causes discomfort in the drinker, this may also prevent migraine patients from drinking alcohol (Quertemont and Didone 2006; Lehner et al. 2024). And the ADH2 His allele (ADH2*2) is rare in non‐Asian populations (He et al. 2015). This may support the avoidance of alcohol in migraine patients in the US population.

The exact mechanism by which dietary alcohol intake contributes to headaches is unclear (McMurtray et al. 2013). Alcohol use and headaches do not seem to be adequately explained by the conventional vasodilator‐constrictor theory of migraine; instead, it may be more likely that specific receptors in the brainstem or cerebral cortex are involved (Schramm et al. 2013). For most migraine patients, there are often both short‐term episodes and long‐term intermittent episodes, a condition that can be caused by overstimulation of the brain (Lipton et al. 2014). Therefore, an alternative explanation for our findings is that alcohol intake may reduce migraine attacks, but the exact mechanism is not clear. Short‐term alcohol consumption reduces brain excitability, which inhibits brain function. This phenomenon works by altering the delicate balance between excitatory and inhibitory transmitters. Alcohol may work by inhibiting the transmission of excitatory transmitters, enhancing inhibitory transmitters, or both mechanisms may work synergistically (Valenzuela 1997). There is evidence that young alcoholics have significantly increased gamma‐aminobutyric acid (GABA) concentrations from Day 1 to Day 2 after alcohol consumption (Marinkovic et al. 2022). And, it has also been reported that drinking beer a few days before a headache attack reduces the risk of migraine and headaches and shortens their duration (Hindiyeh et al. 2020; Wöber et al. 2007). Based on the results of these two studies, we can find that there may be a relationship between the frequency of alcohol consumption and migraine (Yokoyama et al. 2012; Kim et al. 2021). This evidence may support our second interpretation of the results, which is that short‐term alcohol consumption achieves a reduction in headache odds by suppressing the brain. However, more detailed and direct evidence regarding whether it works specifically through inhibitory transmitters is still needed. In addition, some evidence is less supportive of this interpretation. Alcohol consumption is greater in Europe than in Asian countries, but alcohol‐induced migraine has been reported more frequently in Europe (Masip and Germà Lluch 2021). However, further investigation is needed, limited by different drinking patterns such as time of alcohol intake and type of alcohol. Our findings fill a gap in the investigation of alcohol and headache in the US population, enriching the sample size and diversity of the study.

For the gender differences present in the results, it may be that women seem to have a different subjective drinking experience because their blood alcohol concentration is higher and lasts longer than that of men. This biological basis may allow women to feel intoxicated when they drink less alcohol, which may be an inhibitory mechanism for them (Erol and Karpyak 2015). For the effect of the relationship between alcohol and headache produced by age, we hypothesize that it may be due to the decreased metabolic level of the elderly, which leads to a longer duration of alcohol in the brain, resulting in a prolonged inhibitory effect on the brain, which makes the frequency of headache less frequent (Błaszczyk et al. 2023) and finally makes it less likely that headache will occur in the older group.

The World Health Organization's report, stating that the safe intake of dietary alcohol is 0 (Peacock et al. 2018), suggests that we should not encourage the consumption of alcohol. In addition, Dr. García‐Azorín's study, which indicated that the frequency of attacks the next day did not increase in patients with migraine who had consumed alcohol, concluded that patients with low‐risk migraine could consume alcohol in moderation (García‐Azorín 2022). This coincides with the findings of Panconesi et al. (2012).

For this study, we used the nationally representative NHANES database with a large sample size. In addition, we adjusted for confounders to provide a full range of considerations. Finally, we conducted subgroup analyses to investigate the relationship between exposure factors and outcomes in populations under different strata to ensure stability of the relationship between exposure factors and outcomes. However, our cross‐sectional study design precludes establishing causality between alcohol intake and migraine. Future prospective or intervention studies are needed to validate these findings. In addition, we could not rule out all potential confounding factors despite considering multiple covariates. Another limitation is that the study focused solely on the US population. Despite subgroup analyses by race and family PIR values, further research is needed to validate these findings in other populations, given the diverse drinking habits influenced by cultural and religious differences worldwide. For instance, religious prohibitions in Iran significantly reduce alcohol consumption among Iranians compared to Europeans (Muñoz et al. 2018). In contrast, historical and cultural practices, such as the longstanding tradition of wine consumption in Europe, contribute to higher alcohol consumption rates in European populations relative to those in many Asian countries (Masip and Germà Lluch 2021; Peacock et al. 2018). Statistics on dietary alcohol intake in the NHANES database are limited due to the lack of information on alcohol type and frequency of consumption. Furthermore, 24‐h dietary recalls via telephone and face‐to‐face interviews may introduce recall bias and limit the conclusiveness of our assessments.

## 5 Conclusion

In conclusion, increased dietary alcohol intake was associated with decreased odds of migraine or severe headaches, particularly in older populations. This suggests that alcohol‐induced migraine may not be as severe as previously thought. Alcohol intake is not universally contraindicated for individuals with migraine. In clinical practice, it is possible to move from total prohibition to precise management. Structured trigger identification through standardized tools (e.g., a 3‐month digital headache diary [Buse et al. 2019; McGinley et al. 2022]) could be considered, which could distinguish true alcohol triggers from coincidental associations (Turner et al. 2019). This approach reduces unnecessary lifestyle restrictions. At the same time, reintroducing a monitoring program for alcohol in patients with unidentified triggers may prevent maladaptive avoidance behaviors from impairing social functioning (Hindiyeh et al. 2020; Ulrich et al. 2024). Public health messages need to be culturally adapted to regional drinking patterns, distinguishing between primary prevention (discouraging alcohol use among non‐drinkers) and secondary prevention (individualized abstinence strategies for current drinkers) (Park et al. 2016; Hajjarzadeh et al. 2022). Ultimately, combining evidence‐based trigger education with quality‐of‐life protection frameworks can ensure that dietary advice neither overemphasizes theoretical risks nor ignores the biopsychosocial complexities of migraine management. However, it is important to emphasize that due to the broader health risks associated with alcohol consumption, it remains appropriate to limit alcohol intake in the migraine population.

## Author Contributions

**Yi Tang**: Methodology; Writing–original draft. **Kangrui Zhang**: Methodology. **Yueyu Zhang**: Data curation; Investigation. **Xinhui Jia**: Methodology. **Jiaxuan Li**: Data curation; Investigation. **Jie Hu**: Data curation; Investigation. **Xun He**: Data curation; Investigation. **Xinyi Chen**: Data curation; Investigation. **Juncang Wu**: Writing–review & editing; Conceptualization; Funding acquisition.

## Ethics Statement

The NCHS Ethics Review Board approved and the study was carried out in compliance with the Declaration of Helsinki for the parts that involved human subjects, human materials, or human data. To take part in this study, the patients/participants gave their written informed consent.

## Conflicts of Interest

The study's authors declare that there were no financial or commercial relationships that may be construed as creating a conflict of interest.

### Peer Review

The peer review history for this article is available at https://publons.com/publon/10.1002/brb3.70400

## Data Availability

Researchers and data users around the world can access the survey data via the Internet (www.cdc.gov/nchs/nhanes/).
